# A role for Separase in telomere protection

**DOI:** 10.1038/ncomms10405

**Published:** 2016-01-18

**Authors:** Francesca Cipressa, Patrizia Morciano, Giuseppe Bosso, Linda Mannini, Alessandra Galati, Grazia Daniela Raffa, Stefano Cacchione, Antonio Musio, Giovanni Cenci

**Affiliations:** 1 Department of Biology and Biotechnology “Charles Darwin” Section of Genetics, SAPIENZA University of Rome, P.le Aldo Moro 5, 00185 Rome, Italy; 2 Istituto Pasteur, Fondazione Cenci-Bolognetti, Viale Regina Elena 291, 00185 Rome, Italy; 3 Istituto di Ricerca Genetica e Biomedica, Consiglio Nazionale delle Ricerche, c/o Area di Ricerca di S. Cataldo Via G. Moruzzi 1, 56124 Pisa, Italy; 4 Istituto Toscano Tumori, Via T. Alderotti 26N, 50139 Firenze, Italy

## Abstract

*Drosophila* telomeres are elongated by transposition of specialized retroelements rather than telomerase activity and are assembled independently of the sequence. Fly telomeres are protected by the terminin complex that localizes and functions exclusively at telomeres and by non-terminin proteins that do not serve telomere-specific functions. We show that mutations in the *Drosophila* Separase encoding gene *Sse* lead not only to endoreduplication but also telomeric fusions (TFs), suggesting a role for *Sse* in telomere capping. We demonstrate that Separase binds terminin proteins and HP1, and that it is enriched at telomeres. Furthermore, we show that loss of Sse strongly reduces HP1 levels, and that HP1 overexpression in *Sse* mutants suppresses TFs, suggesting that TFs are caused by a HP1 diminution. Finally, we find that siRNA-induced depletion of ESPL1, the Sse human orthologue, causes telomere dysfunction and HP1 level reduction in primary fibroblasts, highlighting a conserved role of Separase in telomere protection.

## Abstract (web summary)

*Drosophila* telomeres are elongated by transposition of specialized retroelements rather than telomerase activity. Here, the authors show that Separase is enriched at *Drosophila* telomeres and loss of *Sse*, the gene encoding Separase, leads to telomere defects, suggesting a role for Separase in telomere protection.

## Graphical abstract

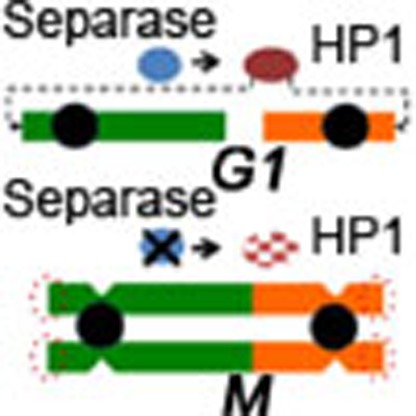

Chromosome ends in eukaryotic cells are protected and replicated by specialized nucleoproteic complexes called telomeres. In most organisms, these complexes comprise short, repetitive G-rich sequences added to chromosome ends by telomerase, a reverse transcriptase with an internal RNA template. Telomeric sequences are bound by specific protein complexes that permit cells to distinguish chromosome ends from DNA break sites^1^,^2^. Excessive telomere shortening or failure in assembling proper telomere protein complexes render chromosome ends dysfunctional, thus limiting proliferative lifespan and leading to genome instability. In *Drosophila*, telomerase is absent and telomere length is maintained by transposition of three specialized retroelements, namely Het-A, TART and TAHRE. However, chromosome end protection is achieved by sequence-independent association of protein complexes that prevent checkpoint activation and end-to-end fusion as in other eukaryotic systems^3^,^4^. Genetic and molecular analyses of genes specified by mutations causing telomeric fusions (TFs) in larval brain cells have thus far identified 11 genes required to prevent TFs in *Drosophila*, which encode three classes of proteins: (i) the telomere-specific terminin components (HOAP, Moi, HipHop and Ver); (ii) DNA repair/checkpoint proteins (ATM and the MRN complex) that are also required for terminin recruitment at telomeres; and (iii) non-terminin capping proteins (Woc, UbcD1, HP1 and Peo) that protect telomeres independently of terminin^3^,^4^,^5^. This suggests that multiple factors contribute to *Drosophila* telomere protection and implies that multiple DNA end-joining pathways may be involved in recognition and processing of unprotected telomeres. With the exception of terminin, all *Drosophila* capping factors are conserved in human and some are shown to play telomere-related function^3^,^4^. Interestingly, the human and mouse homologues (AKTIP and Ft1) of *Drosophila* Peo are indeed required for telomere maintenance^6^. It is therefore conceivable that the identification of additional *Drosophila* genes encoding non-terminin proteins involved in telomere protection might lead to the discovery of novel components of human telomeres.

Here we demonstrate that mutations in the *Drosophila* Separase encoding gene *Sse* lead not only to endoreduplication^7^ but also TFs. In addition, we show that Sse can physically interact with both terminin proteins and HP1. Immunostaining and Chromatin immunoprecipitation (ChIP) analyses revealed that Sse is enriched at telomeres as well. Moreover, although localizations of terminin and Sse are not interdependent, loss of Sse strongly reduces HP1 levels. Overexpression of HP1 in *Sse* mutants suppresses TFs but does not rescue the endoreduplication phenotype, suggesting that TFs seen in *Sse* mutants are due to reduction of HP1 levels. A catalytically inactive Sse fails to restore the HP1 levels and to reduce TFs, indicating that Sse endopeptidase activity is ultimately required for telomere protection. Finally, we provide evidence that small interfering RNA (siRNA)-induced depletion of ESPL1, the Sse human orthologue^8^,^9^,^10^, induces telomere-dysfunction-induced foci (TIFs) and reduces levels of HP1 in human primary fibroblasts (HPFs), suggesting that Separase plays an evolutionarily conserved role in telomere protection.

## Results and Discussion

### *Drosophila Separase (Sse)* mutants exhibit TFs

**Figure 1:**
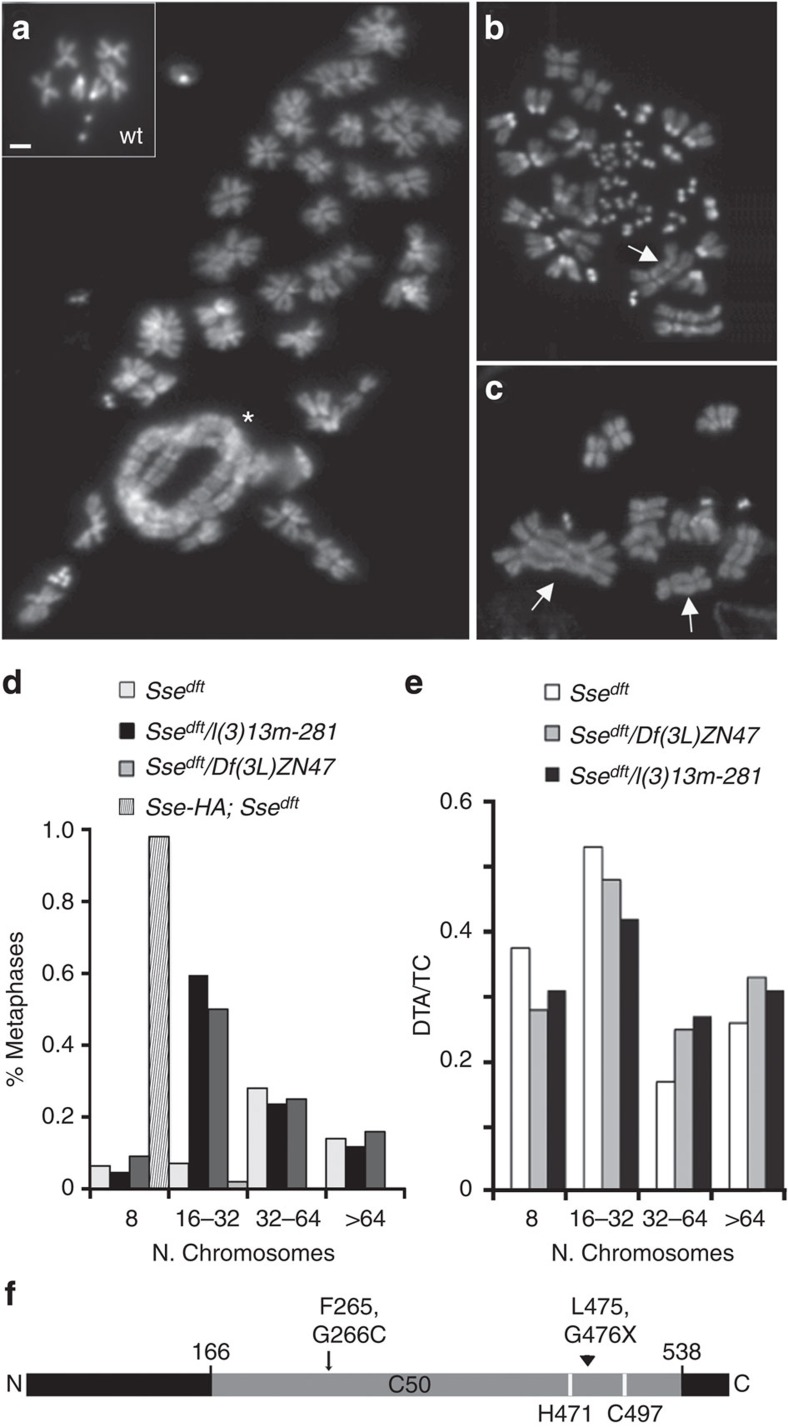
*Drosophila* Separase protects telomeres from fusions. (**a**) DAPI-stained neuroblast metaphases from wild type (inset in **a**) and *Sse* mutant (**a**–**c**) larval brains. (**a**–**c**) Examples of metaphases from *Sse* mutants showing different degrees of endoreduplication; some endoreduplicated chromosomes are fused at their termini generating complex linear (arrows in **b** and **c**) and ring-shaped (asterisk in **a**) multicentric chromosome configurations. It is worth noting that the arrows indicate the most straightforward DTAs. Scale bar, 5 μm. (**d**) Degree of ploidy in endoreduplicated cells of *Sse* mutants. (**e**) Frequency of TFs in *Sse* mutants; DTA/TC is the ratio between the number of DTAs and the *t*otal number of metaphase *c*hromosomes (TC). It is noteworthy that the DTA/TC ratio does not change with the degree of endoreduplication. In wild-type brains, TFs and endoreduplicated metaphases were never observed. (**f**) Schematic representation of *Drosophila* Sse showing the conserved C50 peptidase domain (grey), the invariant Histidine and Cystein residues (white bars), and the 3-bp deletion that replaces F265 and G266 with a C in the *Sse*^*dft*^ mutant (black arrow). This mutation generates a protein that is one amino acid shorter than a wild-type Sse. The arrowhead indicates the 4-bp deletion generating a premature stop codon previously identified in the *13m-281 Sse* mutant allele.

In the course of a functional analysis of the histone H2 variant (H2A.V), we found that a chromosome with a mutation in the *H2A.V* locus (*l(3)05146* obtained from the Bloomington Stock Center) harbours a second site mutation that severely affects larval brain chromosomes. Larvae homozygous for this second site mutation exhibited brains with very few dividing cells (∼3 metaphases per brain); most cells were endoreduplicated, showing bundles of two, four and eight sister chromosomes and ploidy levels ranging from 4n to 32n (Fig. 1). A large number of metaphases analysed contained 64 (16n) or more than 64 chromosomes (Supplementary Table 1), indicating that mutant brains undergo multiple cycles of endoreduplication. We have also found rare chromosome breaks and rearrangements. Interestingly, some endoreduplicated chromosomes were also fused at their ends, giving rise to endoreduplicated multicentric chromosome configurations and rings (Fig. 1). Based on this peculiar phenotype, we named the gene specified by this mutation *diplofusedtelomeres* (*dft*).

Meiotic recombination and deficiency mapping revealed that *dft* maps on 3L chromosome arm and is uncovered by *Df(3L)ZN47*, which removes the 64C–65C polytene region. Complementation analysis between *dft* and mutations in the same region revealed that *dft* is allelic to *l(3)13m-281*, a null mutation in the Separase-coding gene *Sse*^7^,^11^. Heteroallelic combinations between *dft* (hereafter designated as *Sse*^*dft*^) and *l(3)13m-281* displayed the same classes of chromosome phenotypes observed in *Sse*^*dft*^ homozygotes and hemizygotes (Fig. 1 and Supplementary Table 1), suggesting an unanticipated role of *Drosophila* Separase in telomere capping. To measure the frequency of TFs we calculated the ratio between the number of double telomere attachments (DTAs) and the total number of chromosomes (TCs). The analysis of ∼250 *Sse* mutant metaphases showed an average DTA/TC value of ∼0.35. We found that in all mutant combinations analysed, DTAs caused mainly the formation of multicentric and/or ring configuration (∼85%). However, as the complexity of endoreduplicated cells may hinder an exact evaluation of total number of fused chromosomes, we believe that number of fusions is underestimated. Nevertheless, this value did not significantly vary between the diploid metaphases and the endoreduplicated cells of different ploidy (Fig. 1e). This observation, along with the finding that DTAs are also found in euploid cells (Supplementary Fig. 1 and Supplementary Table 1), suggests that telomere fusion in *Sse* mutant brains is independent of endoreduplication.

To confirm that observed chromosome associations are indeed caused by telomere fusions, we sought to verify whether fusion sites contained copies of HeT-A, the most abundant telomeric transposon^12^. Thus, we performed DNA fluorescent *in situ* hybridization with a HeT-A probe mix, which normally recognizes most *Drosophila* telomeres^13^,^14^. The analysis of ∼20 *Sse*^*dft*^ mutant larval brains has revealed the presence of HeT-A-specific signals at fused telomeres that were similar to those observed at free telomeres (Supplementary Fig. 2), indicating that chromosome associations consist of intact telomeric sequence fusions.

Sequence analysis revealed that the Sse-coding region in *Sse*^*dft*^, in addition to containing several nucleotide changes ascribable to natural variations (Supplementary Fig. 3), harbours a 3-bp deletion (TTG) at position 794 from the initiation codon. This deletion causes loss of Phe and Gly at position 265 and 266 of the Sse amino acid sequence, respectively. Both residues are replaced by a Cys at position 266, ultimately giving rise to a polypeptide lacking a single amino acid and which is no longer functional (Fig. 1f). Both the telomere fusion and the endoreduplication phenotypes of *Sse*^*dft*^ mutants were rescued by the expression of a haemagglutinin (HA)-tagged wild-type *Sse* transgene (Fig. 1d,e). Consistent with this result, RNA interference (RNAi)-mediated Sse depletion in larval neuroblasts caused a chromosomal phenotype very similar (although less severe) to that seen in *Sse*^*dft*^mutants (Supplementary Fig. 4 and Supplementary Table 1). We also analysed the expression of the Sse protein in mutant flies using an affinity-purified chicken anti-Sse antibody we have generated. This antibody recognized a band of the expected molecular weight (∼75 kDa; Supplementary Figs 7a and 8f) and this band was drastically reduced in brain extracts from all *Sse* mutant combinations (Supplementary Figs 8f and 9). Thus, our results indicate that *Sse*^*dft*^ is a strong hypomorphic allele of the *Sse* gene, and that *Sse* is required for telomere protection.

Previous studies have shown that Sse requires two additional factors to mediate sister chromatid separation in *Drosophila*: Pimples (Pim) that has functional similarities with securin and Three rows (Thr) that corresponds to the amino-terminal regulatory domain present in Separases of non-dipteran species but not in *Drosophila* Sse^7^. To investigate whether Pim and Thr play roles at telomeres, we depleted each of these Sse-interacting proteins in larval brains using *UAS pim* or *UAS thr RNAi* constructs driven by the *69BGAL4* driver (Supplementary Fig. 12). We used *in vivo* RNAi because both *pim* and *thr* loss-of-function mutations cause embryonic lethality, preventing a reliable cytological characterization of a telomeric phenotype. We found that *pim* and *thr* RNAi brain cells exhibit frequent diplochromosomes but no TFs (Supplementary Fig. 4), indicating that in contrast to Sse neither Pim nor Thr has an obvious role in telomere capping.

It can be argued that the telomeric effects of Sse depletion is due to persistent cohesion of chromosome ends as previously reported in human cells^15^. However, persistent cohesion at telomeres is likely to give rise to sister unions instead of train of chromosomes^15^. Moreover, our analysis on 50 no-colchicine-treated *Sse* mutant metaphases revealed that all chromosomes had separated, uncohesed sister telomeres, suggesting that loss of Sse does not impair resolution of sister telomeres. This observation is in line with the findings that loss of *Drosophila* Wapl, a protein that regulates binding of the cohesin complex to chromosomes during interphase and helps remove cohesin from chromosomes during mitosis, does not affect telomere behaviour^16^,^17^. It is thus conceivable that telomere fusions observed in *Sse* mutants are independent of defective removal of cohesin.

### Sse binds telomeric proteins and localizes at telomeres

**Figure 2:**
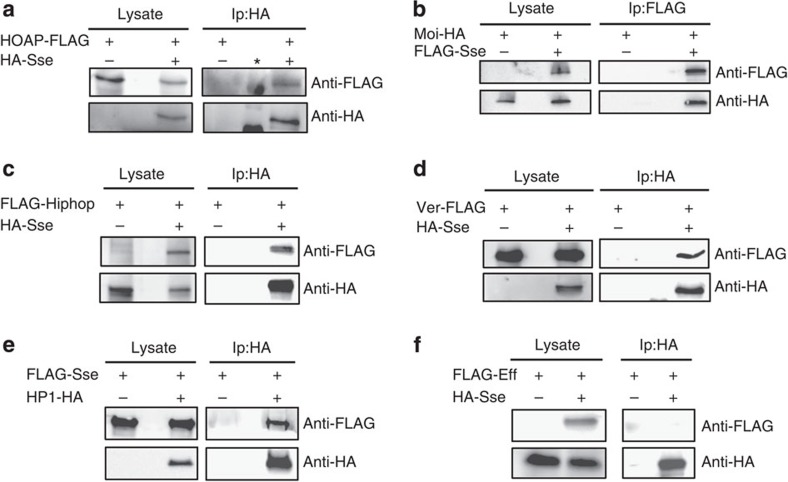
Sse interacts with telomeric proteins. Immunoprecipitation experiments using an anti-Flag affinity gel or an anti-HA affinity matrix from S2 cell extracts co-expressing HOAP-FLAG and HA-Sse (**a**), Moi-HA and FLAG-Sse (**b**), Flag-HipHop and HA-Sse (**c**), Ver-FLAG and HA-Sse (**d**), FLAG-Sse and HA-Hp1 (**e**), and FLAG-Eff and HA-Sse (**f**). As negative control, immunoprecipitations were performed from extracts expressing only the corresponding tagged telomeric protein. It is noteworthy that, with the exception of Eff, Separase precipitates or is precipitated by all telomeric proteins tested. *An aspecific antibody cross-reaction with the protein marker. Lysate, 10% of total protein extracts.

We next investigated whether Sse interacts with terminin proteins. For this purpose, we performed immunoprecipitation assays using extracts from S2 cells expressing FLAG-Sse and HA-Moi, HA-Sse and HOAP-FLAG, HA-Sse and FLAG-Hiphop or HA-Sse and Ver-FLAG. This analysis revealed that Sse co-precipitates with HOAP, Ver, Moi and Hiphop, indicating that it forms a complex with terminin (Fig. 2a–d). The same approach was used to ask whether Sse interacts with some selected non-terminin factors such as HP1 and Eff^4^. We found that tagged-Sse co-precipitated with HP1-HA but not with FLAG-Eff (Fig. 2e,f), indicating that Sse does not generally bind all telomere-capping factors. In addition, HP1-HA is also able to immunoprecipitate endogenous Sse, indicating that this interaction is not due to Sse overexpression (Supplementary Fig. 5). As a complementary approach, we carried out glutathione *S*-transferase (GST)-pulldown experiments from larval brain extracts expressing HA-Sse, which confirm that Sse is precipitated by GST-Ver, GST-Moi, GST-HOAP and GST-HP1 but not by GST-Eff and GST-Peo (Supplementary Fig. 6). Collectively, these results strongly suggest that Sse associates with terminin components and with non-terminin HP1.

**Figure 3:**
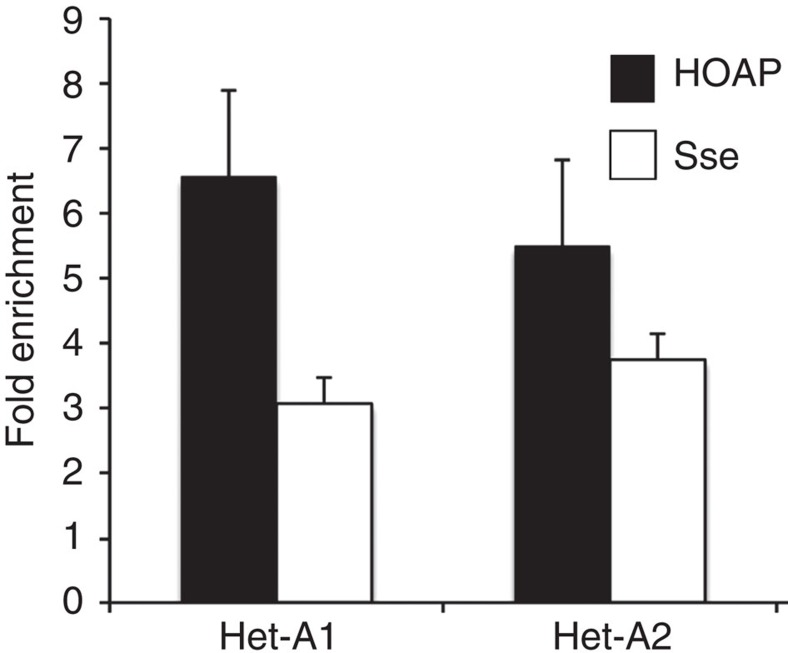
Separase associates with the telomeric transposon *Het-A*. The HOAP and Sse enrichment for Het-A sequences were measured as ChIP-F/input-F/ChIP-N/input-N ratio, where F refers to Flag-HOAP (or -Sse) ChIP fractions and N to negative control (no-Flag, ChIP from untransfected cell extracts). Values were normalized relative to the *1731* non-telomeric transposon used as a standard. Columns indicate fold enrichments for HOAP (black) and Sse (white) at the *Het-A1* and *Het-A2* sequences with respect to their enrichment at the *1731* control sequence. Error bar indicates s.e.m.

We also asked whether Sse was enriched at telomeres. For this purpose, we immunostained polytene and mitotic chromosomes with the same anti-Sse antibody used for western blotting. Interestingly, we found that Sse is expressed in salivary glands, although at low levels, suggesting that it plays a role also in non-cycling cells (Supplementary Fig. 7a). Immunostaining of polytene chromosomes revealed that Sse localizes to many euchromatic regions along all chromosome arms (Supplementary Fig. 7b); 65% (*n*=60) of polytene chromosomes from *Sse*^*dft*^*/Df(3L)ZN47* mutants displayed a strong reduction (80%) of stainining, indicating that the Sse^dft^ mutant protein fails to properly localize on chromatin (Supplementary Fig. 7c). About 70% of the Sse signals corresponded to interbands that are not stained by 4,6-diamidino-2-phenylindole (DAPI), whereas the remaining 30% appeared to coincide with thin bands that were weakly stained by DAPI. In addition, we found that Sse associated also with telomeric regions in ∼1/3 of chromosome tips (*n*=120; Supplementary Fig. 7b). This staining pattern suggests that *Drosophila* Sse binds chromatin similar to its human counterpart^18^. Immunostaining of mitotic cells from brain squashes with our anti-Sse antibody resulted in rather diffuse and punctate pattern, which could not be improved using different fixation procedures. Nevertheless, we found that 40% (*n*=200) of wild-type mitotic telomeres displayed Sse signals that precisely co-localized with HOAP signals, which are specifically associated with telomeres^19^ (Supplementary Fig. 8a,e). Telomeric Sse signals were also detected in HA-Sse-expressing brain metaphases immunostained with an anti-HA antibody (Supplementary Fig. 8b,e) or with another anti-Sse antibody described in a previous study^7^. Thus, biochemical results suggest that Sse binds telomeres, and cytological observations along with quantification of signal intensity (Supplementary Fig. 8d) indicate that Sse is sixfold more enriched at telomeres than at chromosome arms. To confirm that Sse associates with telomeric chromatin, we performed ChIP and real-time PCR, to measure the enrichment of Sse at telomeres of S2 cells expressing FLAG-Sse. As a positive control, we measured the telomeric enrichment of terminin HOAP-FLAG, which is known to be strongly associated with telomeric chromatin^20^. By using two primer pairs (Het-A1 and Het-A2) from the 3′-untranslated HeT-A region, we observed significant enrichments for both HOAP (∼7-fold and∼6-fold enrichment for Het-A1 and Het-A2, respectively) and Sse (∼3-fold and∼4-fold enrichment for Het-A1 and Het-A2, respectively) when compared with the non-telomeric transposon *1731* (Fig. 3)^21^. These results, which are consistent with our cytological and biochemical observations, confirm that Sse is able to associate to chromosome ends.

### Sse regulates HP1 levels and localization

**Figure 4:**
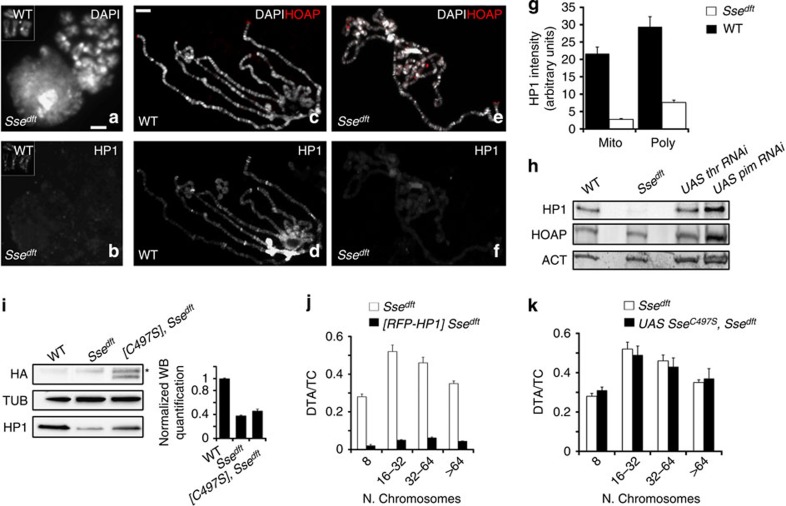
HP1 loading on chromatin might depend on Sse catalytic activity. (**a**,**b**) An *Sse* mutant metaphase stained for DNA (DAPI) (**a**) and HP1 (**b**); the insets show a wild-type metaphase. Scale bar, 5 μm.(**c**–**e**) Polytene chromosomes from wild-type (**c**,**d**) and *Sse*^*dft*^ mutant salivary glands (**e**,**f**) stained with DAPI and immunostained for HOAP (red) and HP1. It is worth noting that in *Sse* mutant chromosomes HOAP localizes normally at telomeres, while HP1 localization is strongly reduced. Scale bar, 10 μm. (**g**) Quantification of HP1 intensity from WT and *Sse*^*dft*^ mitotic (Mito) and Polytene (Poly) chromosomes. The intensity values, which have been measured with the Image J software, are indicated as arbitrary units. Only polytene chromosomes exhibiting a strong reduction of Hp1 staining have been considered for this analysis. (**h**) Western blot from wild type, *Sse*, *thr* and *pim* RNA-interferred extracts showing that loss of Sse specifically reduces HP1 level; actin is used as a loading control. (**i**) Western blotting of brain extracts from wild type, *Sse* mutants and *Sse* mutant expressing a HA-tagged catalytically inactive (C497S) Sse protein. *A nonspecific band. Quantification from three different western blottings of the HP1 levels with respect to tubulin (loading control) shows that the expression of catalytically inactive Sse fails to restore normal HP1 levels. (**j**) HP1 overexpression drastically reduces the TFs frequency in *Sse* mutants. Error bar indicates s.e.m. (**k**) Frequency (±s.e.m) of TFs in *Sse* mutant neuroblasts that overexpress Sse^C497S^.

We then sought to understand whether Sse is required for proper localization of terminin and HP1 at telomeres. We found that loss of Sse does not affect telomeric localization of HOAP (in both mitotic and polytene chromosomes) and Ver (in polytene chromosomes; Fig. 4e and Supplementary Figs 10 and 11), indicating that Sse is not required for recruitment of terminin at telomeres. In contrast, HP1, which is normally enriched in all heterochromatic regions and telomeres^22^, appeared drastically reduced in mitotic and polytene chromosomes of *Sse* mutants compared with wild-type cells. In 90% of mutant metaphases (*n*=40) HP1 failed to localize at both telomere and pericentric chromatin (Fig. 4a,b,g), and, consistent with this finding, 76% (*n*=50) of *Sse* mutant polytene chromosomes showed a strong (∼70%) reduction of HP1 immunostaining (Fig. 4f,g). Thus, mutations in *Sse* strongly reduce the amount of HP1 associated with telomeres or heterochromatin. Western blot analysis revealed that the HP1 level, but not that of HOAP (as expected from immunostaining results), is drastically reduced in *Sse* mutant brain extracts (Fig. 4h). However, *Sse* mutant brains displayed the same level of HP1-encoding *Su(var)205* messenger RNA as wild-type controls (Supplementary Fig. 12). As expected, RNAi-mediated Pim (or Thr) depletion neither reduced the *Su(var)205* mRNA level nor the HP1 amount associated with the chromosomes (Fig. 4h and Supplementary Fig. 13). Collectively, these results indicate that Sse knockdown specifically affects HP1 stability reducing the amount of HP1 associated to heterochromatin and telomeres.

Previous work has shown that mutations in *Su(var)205* cause frequent TFs^22^. Thus, the TFs observed in *Sse* mutants might be the consequence of the HP1 reduction. To test this hypothesis, we measured the frequency of TFs in *Sse* mutant larvae carrying an *RFP-Su(var)205/HP1* transgene that yields ∼3-fold increase in the HP1 protein level. These mutant larvae exhibited a drastic reduction in the DTA/TC ratio (0.04 versus 0.40; Fig. 4j and Supplementary Table 1), although the frequency of endoreduplicated cells did not vary with respect to the *Sse* mutant. We thus conclude that HP1 reduction in *Sse* mutants leads to TFs but not to endoreduplication, confirming that TFs and endoreduplication are independent outcomes of mutations in the *Sse* gene. The finding that the telomeres in *Sse* mutants undergo fusion, although they retain terminin, is not unexpected, as it is well known that HP1 protects telomeres independently of terminin^3^,^19^,^23^,^24^.

We also asked whether the catalytic activity of Sse is required for telomere protection. We thus drove the expression in *Sse* mutant brains of the *UAS-HA-Sse*^*C497S*^ transgene, which encodes a catalytically inactive *Sse* in which the predicted catalytic cysteine residue at position 497 is replaced by a serine^7^. The expression of Sse^C497S^ in *Sse*^*dft*^ mutant brains failed to restore the wild-type HP1 levels, to reduce the frequency of TFs and to suppress endoreduplication (Fig. 4i,k and Supplementary Table 1), whereas a wild-type Sse rescued all *Sse*^*dft*^ phenotypes, including HP1 reduction (Supplementary Fig. 14). These observations indicate that both HP1 regulation and telomere capping depend on the Sse catalytic activity.

### The Sse human orthologue ESPL1 plays a role at telomeres

**Figure 5:**
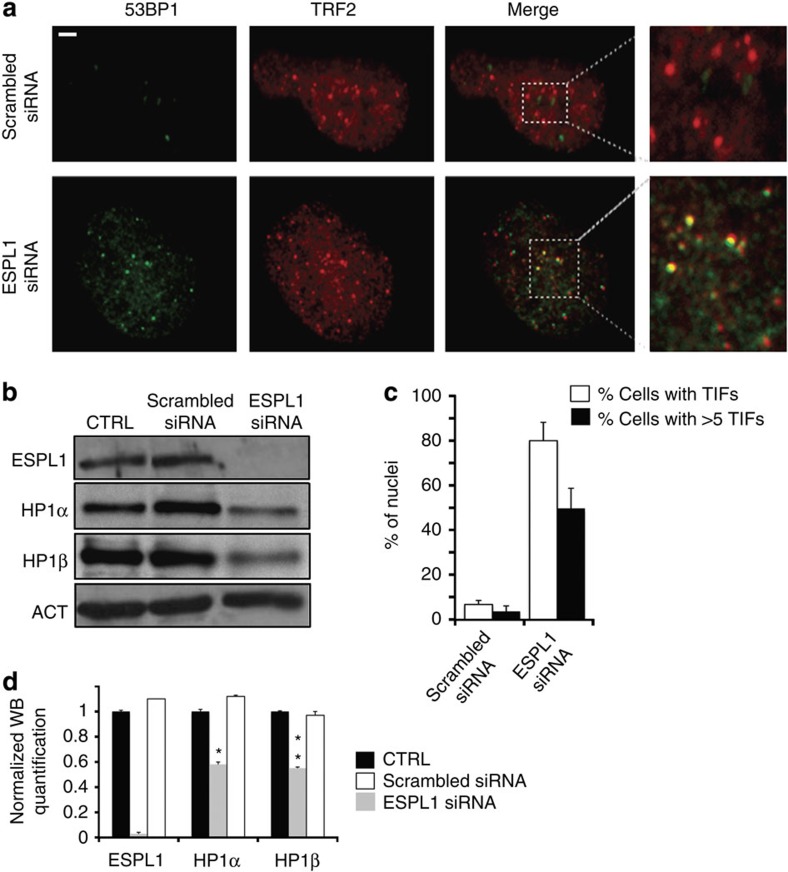
RNAi-mediated depletion of human separase (*ESPL1)* induces TIFs. (**a**) Control scrambled siRNA- (control) and *ESPL1 siRNA*-treated fibroblasts stained with anti-p53-binding protein 1 (53BP1; green) and anti-TRF2 (red). It is noteworthy that in ESPL1 siRNA-treated cells, 53BP1 signals frequently overlap with TRF2 signals marking the TIFs. Scale bar, 5 μm. (**b**) Western blot analysis of extracts from untreated (ctrl), scrambled siRNA- and *ESPL1 siRNA-*treated human primary fibroblasts. Actin was used as a loading control. (**c**) Average frequency (empty columns±s.e.m) of 53BP1 foci that co-localize with TRF2 (TIFs; ∼50 nuclei examined) and frequencies of nuclei containing at least 5 TIFs (black columns). (**d**) Quantification of HP1α and HP1β levels with respect to actin (loading control) from four different western blottings that shows the reduction of levels of both HP1 proteins after ESPL1 loss (Student's *t*-test, *P<0.05; ** P<0.01).

*Drosophila* Sse and its human orthologue ESPL1 (Extra Spindle Poles-like 1 protein) share a substantial level of homology and functional analogy in the peptidase domain typical of cystein peptidase family C50 (ref. 25). Moreover, heterologous interactions between *Drosophila* Sse and human Securin have been previously demonstrated^26^, suggesting that *Drosophila* and human Sse-interacting proteins adopt a related tertiary structure. We thus asked whether loss of ESPL1 affects telomere stability in human cells. Depletion of ESPL1 by siRNA in HPFs caused the formation of telomere-associated DNA repair foci, dubbed TIFs (Fig. 5a). About 80% of *ESPL1* siRNA-treated HPFs (*n*=100) showed co-localization of the DNA repair p53-binding protein 1 with the telomeric protein TRF2 in TIFs and 50% of these cells exhibited more than 5 TIFs. In contrast, only 7% of mock-treated HPFs (*n*=100) showed TIFs (Fig. 5c). In addition, western blotting analysis on total protein extracts from *ESPL1* siRNA fibroblasts showed a substantial reduction (∼40%) of both HP1α and HP1β, the human counterparts of *Drosophila* HP1 (Fig. 5b,d)^27^. Altogether, these observations suggest that human separase positively regulates the stability of HP1 and telomere protection.

Recent work has shown that separase, in addition to mediating sister chromatid separation, is also needed for a wide range of mitotic events, including spindle dynamics, centriole duplication and cytokinesis^28^,^29^,^30^,^31^,^32^,^33^,^34^. Our data provide the first evidence that separase regulates HP1 stability and telomere capping in both flies and humans. Reduction of HP1 levels in human cells has been associated with impaired telomere length maintenance^35^,^36^. Our results show that regulation of human HP1 may be also important for proper telomere capping. Interestingly, the Sse telomeric function relies on its catalytic activity, which is also important for resolution of centromeric cohesion at the metaphase-to-anaphase transition. We have previously shown that the spindle assembly checkpoint component BubR1 associates with uncapped *Drosophila* telomeres, and that its accumulation at telomeres triggers the spindle assembly checkpoint response^37^. Our data on Sse provide additional evidence of a cross-talk between centromeric and telomeric proteins, and suggest that these proteins may interact in several cellular processes.

## Methods

### *Drosophila* strains

The *l(3)05146* mutant line, the *Df(3L)ZN47* deficiency that uncovers *Sse* and the *P [RFP-HP1]* line were obtained from the Bloomington Stock Center. The *UAS Sse RNAi* (v45092), *UAS pim RNAi* (v100534) and *UAS thr RNAi* (v48343) lines, as well as the *69B GAL4* and *Eyeless GAL4* drivers (which express GAL4 in the brains and salivary glands, respectively) were obtained from the Vienna *Drosophila* RNAi center. The *P[w*^*+*^*, UAS-6HA-Sse]* and *P[w*^*+*^*, UAS-6HA-Sse* *C497S]* transgenic lines^7^ are a gift of C. Lehner and are inserted into the X and the second chromosome, respectively. The *l(3)13m-281 Sse* allele^11^ was kindly provided by M. Gatti. The *[RFP-HP1] Sse*^*dft*^- and *69B GAL4 Sse*^*dft*^-bearing chromosome were obtained by recombination and balanced over *TM6c*. The *cav*^*1*^, *Su(var)205*^*05*^, *Su(var)205*^*04*^, *moi*^*1*^ and *ver*^*1*^ mutations were described previously^19^,^22^,^23^,^24^.

To express 6HA-Sse or 6HA-Sse ^C497S^ in larval brains, *P[w*^*+*^*, UAS-6HA-Sse]* or *P[w*^*+*^*, UAS-6HA-Sse* *C497S]* females were crossed to *69B Gal4* males. Expression of 6HA-Sse or 6HA-Sse^C497S^ in *Sse*^*dft*^ mutant brains was obtained by crossing *P[w*^*+*^*, UAS-6HA-Sse]/P[w*^*+*^*, UAS-6HA-Sse]*; *MKRS/TM6c* and *w/w*; *P[w*^*+*^*, UAS-6HA-Sse* *C497S]/P[w*^*+*^*, UAS-6HA-Sse* *C497S]*; *MKRS/TM6c* to *FM7-GFP/Y*; *69B GAL4 Sse*^*dft*^*/TM6B* and *w/Y*; *Sco/CyO-GFP*; and *69B GAL4 Sse*^*dft*^*/TM6B* males, respectively. F1 *P[w*^*+*^*, UAS-6HA-Sse]/FM7-GFP*; *69B GAL4 Sse*^*dft*^*/TM6c* (or and *w/w; P[w*^*+*^*, UAS-6HA-Sse* *C497S]/CyO-GFP*; *69B GAL4 Sse*^*dft*^*/TM6c*) females were then crossed to F1 *P[w*^*+*^*, UAS-6HA-Sse]/Y*; *69B GAL4 Sse*^*dft*^*/TM6c* (or *w/Y; P[w*^*+*^*, UAS-6HA-Sse* *C497S]/CyO-GFP*; *69B GAL4 Sse*^*dft*^*/TM6c)* males, to establish the *P[w*^*+*^*, UAS-6HA-Sse]/P[w*^*+*^*, UAS-6HA-Sse]* (or *P[w*^*+*^*, UAS-6HA-Sse* *C497S]/P[w*^*+*^*, UAS-6HA-Sse* *C497S]); 69B GAL4 Sse*^*dft*^*/TM6c* stock.

RNAi-mediated depletion of Sse, Pim and Thr in larval brains was obtained by crossing *UAS SseRNAi*, *UAS pim RNAi* or *UAS thr* RNAi to *69B GAL4* or *Eyeless 69B GAL4 Sse*^*dft*^*/TM6c* expressing flies.

Information on the genetic markers and balancers used in this study is available at Flybase ( http://flybase.bio.indiana.edu/). Stocks were maintained and crosses were made on standard *Drosophila* medium at 25 °C.

### Anti-Sse antibody production and purification

The anti-Sse antibody was produced by the GeneTel (Madison, WI) antibody production service by immunization of two hens with a synthetic peptide designed on the following Separase epitope: CIKGKDETTPTMNDQPN (aa574-589). The antibody was then isolated from eggs and enzyme-linked immunosorbent assay tested for antibody response. For the purification, 0.5 mg of GST-Sse protein was run on acrylamide/bisacrylamide gel and blotted onto a nitrocellulose membrane. After blocking with 3% BSA in TBS, the membrane was incubated with 22 mg of crude IgY anti-Sse in TBS and incubated overnight on a shaking platform at 4 °C. Membrane was washed three times with the washing solution 1 (50 mM Tris-HCl pH 7.5, 500 mM NaCl) and with washing solution 2 (50 mM Tris-HCl pH 7.5, 100 mM NaCl). Bound antibody was eluted adding 1 ml of 100 mM glycine pH 2.5 on a rocking platform for 30 min and neutralized with 100 μl of 1 M Tris pH 8. Purified antibody was added with 50% glycerol ant stored at −20 °C.

### *Drosophila* chromosome cytology and immunostaining

DAPI-stained colchicine-treated larval brain chromosomes were prepared as previously described^38^. To measure the frequency of TFs we used a DTA/TC ratio where DTA indicates a DTA (involving sister telomeres from different chromosomes) and TC, which specifies the TC (8, 16–32, 32–64 or >64 chromosomes) contained in the selected metaphase. Immunostaining of mitotic metaphase were obtained by dissecting larval brains in 0.7% sodium chloride. Brains were then incubated 45 min with 10^−5^ M colchicine and, after a 7-min treatment with hypotonic solution (0.5% sodium citrate), fixed for 7 min with 1.7% formaldehyde, 45% acetic acid, squashed in the same fixative and then frozen in liquid nitrogen. After flipping the coverslip off, slides were soaked in cold TBS, washed twice in TBS-Tween 0.1% for 5 min and incubated overnight at 4 °C with the appropriate antibodies. Immunostaining of polytene chromosomes were performed by dissecting and fixing salivary glands from third instar larvae following the same procedure used for the brains, with the exception of colchicine and hypotonic treatment, which was omitted. For anti-Sse immunostaining, after fixation and before immunostaining, slides were immersed for 30 min at 90 °C in a slide jar containing 50 ml of Antigen Retrieval Buffer (Leica). For antibody immunostaining, brain and polytene squashes were incubated with rabbit anti-HOAP (1:100), chicken anti-Sse (1:50), rat anti-HA (1:10; Covance MMS-101P), mouse anti-HP1 (1:10), rabbit anti GFP (1:10; kindly provided by Gianluca Cestra) antibodies. Slides were then washed twice in TBS-Tween 0.1% for 15 min and incubated in a humid chamber for 2 h at room temperature with fluorescein isothiocyanate-conjugated goat anti-mouse 1:20 (Jackson Laboratories 115-095-062), AlexaFluor 555-conjugated donkey anti-rabbit (Invitrogen ab150062) 1:200, anti-rat IgG (H+L) 1:20 (Alexa Fluor 647A-21247) or anti-Chicken IgG (H+L) 1:100 (Alexa Fluor 488 ab150169) antibodies. After mounting the slides in Vectashield medium H-1200 with DAPI to stain DNA, both brain and salivary gland preparations were analysed using a Zeiss Axioplan epifluorescence microscope (CarlZeiss, Oberkochen, Germany), equipped with a cooled CCD (charge-coupled device camera; Photometrics, Woburn, MA). Greyscale digital images were acquired as separate files, which were converted to .psd format, pseudocoloured and merged.

For the quantification of fluorescence intensity after anti-Sse and anti-HP1 (in mitotic and polytene nuclei), and anti-HA (in mitotic nuclei) immunostaining, we used the ImageJ software (National Institute of Mental Health, Bethesda, Maryland, USA). Two telomeric and non-telomeric chromosome regions of similar length in mitotic cells and three different chromosome arm regions of similar length in polytene nuclei were selected. In both cases, we quantified the fluorescence of selected areas and the fluorescence of a close chromosome-free region to correct for background fluorescence. For each genotype, the quantification was carried out on at least 15 nuclei.

### Fluorescent *in situ* hybridization

For probe preparation, a mixture of the 2-kb ApaI fragment of the 3′-untranslated region and the 23Zn-1 fragment containing ORF1+ORF2 of HeT-A^39^,^40^ was labelled with digoxigenin-11-dUTP (DIG-Nick Translation Mix, Roche) following the manufacturer's instructions. Three micrograms of sonicated salmon sperm DNA and 80 ng of labelled probe per slide were ethanol precipitated and resuspended in 50% formamide, 10% dextran sulfate and 2 × SSC. Chromosome preparations were dehydrated by sequential immersion in 70%, 90% and absolute ethanol, and then denaturated 2 min at 70 °C in 70% formamide and 2 × SSC. After sequential immersion in cold 70%, 90% and absolute ethanol, slides were incubated with 10 μl of probe at 37 °C in wet chamber. After overnight incubation, slides were whashed three times at 42 °C with 50% formamide and 2 × SSC, followed by three whashes at 60 °C in 0.1 × SSC. Chromosomes preparations were blocked 30 min at 37 °C in 3% BSA, 0.1% Tween 20 and 4 × SSC, and then incubated with 1:50 anti-digoxigenin (Roche 11207750910) dilution in 0.1% BSA, 0.1% Tween-20 and 4 × SSC for 30 min at 37 °C. After three washes at 42 °C in 4 × SSC and 0.1% Tween-20, slides were air dried, mounted in Vectashield medium H-1200 with DAPI and analysed using a Zeiss Axioplan epifluorescence microscope equipped with a cooled CCD camera (Photometrics).

### Production and purification of recombinant proteins

To obtain GST-Sse, GST-Pim, GST-Moi, GST-HOAP, GST-Ver and GST-HP1 fusion proteins, the corresponding full-length complementary DNAs were cloned in the pGEX-6P1 vectors as described previously^24^. Bacterially expressed GST fusion proteins were purified by incubating crude lysates with glutathione sepharose beads (Qiagen) as recommended by the manufacturer.

### GST pulldown and western blotting

To obtain extracts for GST-pulldown and western blot analysis, dissected third instar larval brains were lysed in an ice-cold buffer containing 20 mM Hepes KOH pH 7.9, 1.5 mM MgCl_2_, 10 mM KCl, 420 mM NaCl, 20 mM NaF, 10 mM Na_3_VO_4_, 10 mM BGP, 10 mM phenylmethyl sulfonyl fluoride, 0.1% NP40 and 1 × protease inhibitor cocktail (Roche). Human protein extracts were prepared from primary fibroblasts and HeLa cells expressing HOAP-HA and Moi-HA, collected after 72 h and lysed in an appropriate lysis buffer (20 mM Tris pH 8.0, 420 mM NaCl, 1 mM MgCl_2_, 1 mM dithiothreitol, 0.1% NP40 and protease inhibitor cocktail (Roche)). GST-pulldown assays were carried out using protein extracts from ∼50 brains or 4 μg of recombinant proteins. Protein extracts or recombinant protein (either bacterially expressed or purified from human cells) were incubated with 2 μg of each GST fusion protein bound to sepharose beads for 1 h at 4 °C in an appropriate incubation buffer (20 mM Hepes KOH, 20 mM NaF and 0.8% NP40) Sepharose-bound GST proteins were collected by centrifugation and washed in 20 mM Hepes KOH, 20 mM NaF and 0.8% NP40.

For immunoblotting, SDS–polyacrylamide gels were electroblotted on a nitrocellulose membrane (Bio-Rad) in the transfer buffer (390 mM NaH_2_PO_4_H_2_O and 610 mM Na_2_HPO_4_2H_2_O). Membranes were blocked in 5% low-fat dry milk and then probed with appropriate primary antibody. All blots were developed by the ECL or ECL Plus method (Amersham Biosciences) or Pico and West Fempto ECL (Thermo Scientific). Signals were detected with the ChemiDoc scanning system (Bio-Rad, Hercules, CA). We used the following primary antibodies: monoclonal mouse anti-HP1 (C1A9 (ref. 41), 1:500), chicken anti-Sse (1:1,000), anti-HA horseradish peroxidase (HRP) conjugated (1:500; Roche 12013819001), anti-FLAG HRP conjugated (1:3,000; Sigma A8592), rabbit anti-HOAP (1:3,000), anti-actin HRP conjugated (1:5,000; Santa Cruz Biotechnology SC-1615), anti-Tubulin (1:100,000; Sigma T6199), goat anti-HP1α and anti-HP1β (1:500; Abcam ab9057 and ab10478), and rabbit anti-ESPL1 (1:500; Santa Cruz sc-25839). Secondary antibodies were as follows: sheep anti-mouse IgG HRP conjugated (1:5,000 NA931V) and donkey anti-rabbit IgG HRP conjugated (1:5,000 NA934), both from Amersham Biosciences, rabbit anti-chicken IgG HRP conjugated (1:200,000 Sigma A162P) and rabbit anti-goat IgG HRP conjugated (1:5,000, Novex A16136). Quantification of band intensities was obtained by using the densitometry software ImageJ. Uncropped scans of the most representative blosts are supplied in the Supplementary Information (Supplementary Figs 15–17).

### Cell transfection and immunoprecipitation

All transfections were carried out in S2 cells cultured at 25 °C in Schneider's *Drosophila* Medium (Sigma), which was supplemented with 10% heat-inactivated fetal bovine serum (Invitrogen). To obtain HOAP-FLAG-, Ver-FLAG-, FLAG-HipHop- and Sse-FLAG-expressing S2 cells, *cav*, *ver*, *hiphop* and *Sse* cDNAs were cloned in the *pAWF* (or *pAFW*) vector (DGRC) in frame with the FLAG-encoding sequence. For the expression of Moi-HA, HA-Sse, HA-Eff and Hp1-HA, the *moi*, *Sse*, *eff* and *Su(var)205* full-length cDNAs were cloned in *pAHW* (or *pAWH*) vector (DGRC). All constructs were used to transfect (singularly or in combination) 6 × 10^6^ S2 tissue culture cells using Effectene reagent (Qiagen) and cells were harvested 72 h after transfection, for immunoprecipitation and ChIP experiments. For immunoprecipitation experiments, control and transfected S2 cells were washed in cold PBS and homogenized in lysis buffer containing 50 mM Tris HCl pH 7.8, 150 mM NaCl, 0.1% NP 40, 1% Triton and protease inhibitor cocktail (Complete, Roche). Cell extracts were spun at 15,000*g* for 15 min at 4 °C and supernatants incubated for 2 h at 4 °C with a 30-μl packed volume of anti-flag affinity gel (Sigma) or anti-HA affinity matrix (Roche). The resin was washed three times with lysis buffer and proteins eluted with Laemmli buffer.

### ChIP assay

For ChIP analysis, 2.4 × 10^7^ *Drosophila* S2 cells were cross-linked in 1% formaldehyde for 10 min at room temperature. Cells were then washed with cold PBS and lysed in 1% SDS, 50 mM Tris-HCl pH 8.0 and 10 mM EDTA. Lysates were sonicated chromatin fragments about 500 bp long. Chromatin was diluted 1:10 with 1.1% Triton X-100, 2 mM EDTA, 150 mM NaCl and 20 mM Tris-HCl pH 8.0 and precleared with a 50% salmon sperm DNA/protein A agarose slurry (Millipore). Precleared samples were then incubated with a 30-μl packed volume of anti-flag M2 affinity gel (Sigma) overnight at 4 °C. Immunoprecipitates were washed first with a low-salt buffer (0.1% SDS, 1% Triton X-100, 2 mM EDTA, 20 mM Tris- HCl pH 8.0 and 150 mM NaCl), then with a high-salt solution consisting of 0.1% SDS, 1% Triton X-100, 2 mM EDTA, 20 mM Tris-HCl pH 8.0 and 500 mM NaCl and finally with 0.25 M LiCl, 1% Nonidet P-40, 1% sodium deoxycholate, 1 mM EDTA and 10 mM Tris-HCl pH 8.0 followed by two washes with 10 mM Tris-HCl pH 8.0 plus 1 mM EDTA. Chromatin was eluted with 250 μl of 1% SDS and 0.1 M NaHCO_3_. After adding 20 μl of 5 M NaCl, cross-links were reversed for 4 h at 65 °C. Samples were supplemented with 20 μl of 1 M Tris-HCl pH 6.5, 10 μl of 0.5 M EDTA, 20 μg of RNase A and 40 μg of proteinase K, and incubated 1 h at 45 °C. DNA was then recovered by phenol/chloroform extraction and ethanol precipitation. Input and immunoprecipitated material were analysed using Sso Advanced Universal SYBR green supermix (Bio-Rad) on a 7300 real-time PCR system (Applied Biosystems) using the following primers:

*Het-A1*: forward 5′-ACCATAATGCCAACAGCTCC-3′ and reverse 5′-AGCCAGCATTGCAGGTAGTT-3′;

*Het-A2*: forward 5′-CTGAGGCCTCCAAAGACTTG-3′ and reverse 5′-AATCATATTGCGCGGTTTGT-3′;

*1731:* forward 5′-ATGTTTGTGGAAGGTGGTTTCAGG3′ and reverse 5′-GCTTTTTCATCTTGGGATTGCC-3′.

### RNA isolation and semi-quantitative reverse transcriptase–PCR

Total RNA was isolated from wild-type (Oregon R), RNAi lines and mutant larvae using the RNeasy Mini Kit (Qiagen). Fifty nanograms of RNA were reverse transcribed and amplified using Access RT–-PCR System kit (Promega). The *rp49* gene was used as an internal control. We used the following forward and reverse gene-specific primers:

*Sse:* forward 5′-GATTTAGGCGAGTGGTAACCGT-3′ and reverse 5′-ACTGGGAGAGCCAGTACTCAAA-3′;

*thr:* forward 5′-ATGTCTACTGATATAGCCACCCAGC-3′ and reverse 5′-CGCACTAGCTTAATGATCTCCACA-3′;

*pim:* forward 5′-ATGCGTCGACTCATGGATCAGATTTTAAACAAG-3′ and reverse 5′-ATGCGCGGCCGCCTAAAATAGAACATCAATGCCTT-3′;

*Su(var)205:* forward 5′-AGTGACGGGGATCCATGGGCAAGAAAATCGACAACC-3′ and reverse 5′-AGTGACGGGAATTCTTATATCAGAGTACCAGGATAGGC-3′;

*rp49:* forward 5′-ATCGGTTACGGATCGAACAA-3′ and reverse 5′-GACAATCTCCTTGCGCTTCT-3′.

The PCR products were analysed by 1% agarose gel electrophoresis. Band intensities were quantified using Quantity One 1-D Analysis Software (Bio-Rad).

### TIF analysis

Human fibroblasts for TIF analysis were fixed with 3.7% formaldehyde and slides were incubated with rabbit anti-TRF2 (1:100; Novus NB110-57130) and mouse anti-p53-binding protein 1 (1:200; Upstate 05-725). Secondary antibody incubation was carried out at room temperature for 2 h, using fluorescein isothiocyanate-conjugated goat anti-mouse (Jackson Laboratories 115-095-062) and AlexaFluor 555-conjugated donkey anti-rabbit (Invitrogen ab150062).

## Additional information

**How to cite this article:** Cipressa, F. *et al.* A role for Separase in telomere protection. *Nat. Commun.* 7:10405 doi: 10.1038/ncomms10405 (2016).

## Supplementary Material

Supplementary Information Supplementary Figures 1-17 and Supplementary Table 1
