# Supplementary material for: A role for Separase in telomere protection
Source: Nat Commun. 2016 Jan 18;7:10405. doi: 10.1038/ncomms10405 (PMC4735636; doi:10.1038/ncomms10405)
Supplement: Supplementary Information — Supplementary Figures 1-17 and Supplementary Table 1 [file ncomms10405-s1.pdf]

Supplementary Figures

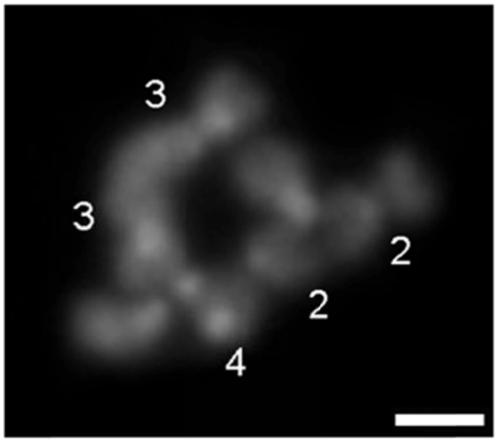

Supplementary Figure 1. ***Sse*^{dft} mutant euploid cells exhibit telomeric fusions**. DAPI stained diploid female metaphase from *Sse*^{dft} mutant brain showing DTAs that involve the 2-2-4 and 3-3 chromosomes. Scale bar, 5 µm.

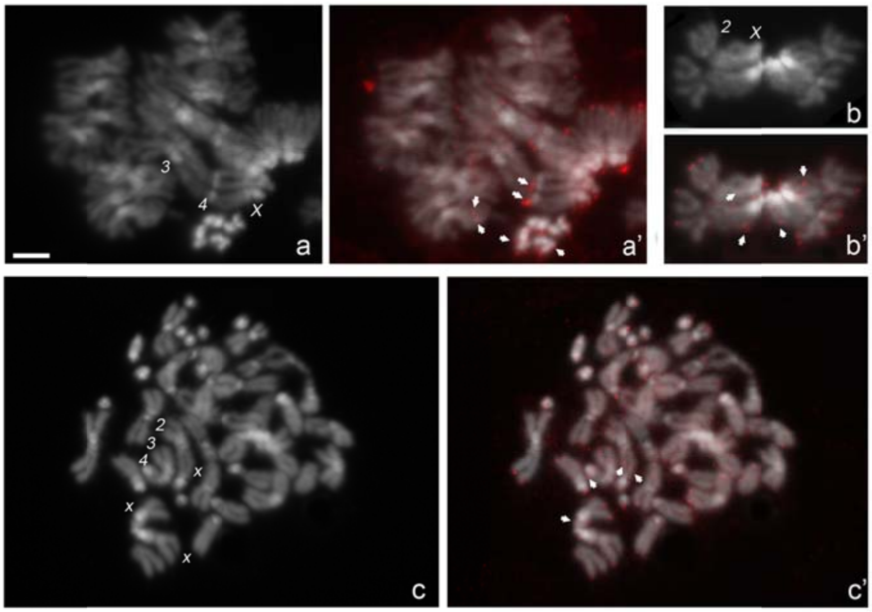

Supplementary Figure 2. **Fluorescent *in situ* hybridization with HeT-A telomeric probe to *Sse*^{dft} mitotic chromosomes**. Het-A probe (red) labels free and fused (arrows in a', b', c') chromosome ends. Small letters and numbers in DAPI stained mutant metaphases (a,b and c) indicate chromosomes involved in telomeric associations. (c,c') Example of a rare *Sse*^{dft} polyploid cell without endoreduplicated chromosomes exhibiting telomere fusions with intact HeT-A signals. Scale bar, 5 µm.

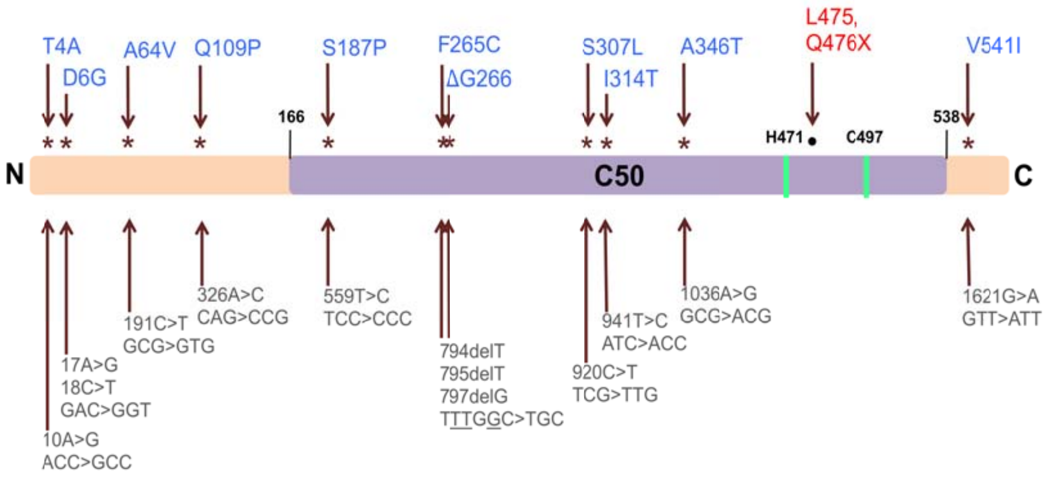

Supplementary Figure 3. Sequence analysis of *Sse*^{dft} mutant locus. Schematic representation of *Drosophila* Sse showing the conserved C50 peptidase domain (magenta), the invariant Histidine and Cystein residues (green bars) and all putative polymorphisms (with respect to the standard gene sequence reported in FlyBase) identified in the *Sse*^{dft} mutant during this study. With the exception of the Glycine deletion residue at position 266, all single nucleotide changes (indicated in gray) generate missense mutations (blue), that are ascribable to natural variation according to the Drosophila Population Genome Project (DPGP). The 4bp deletion generating a premature stop codon previously identified in the *13m-281 Sse* mutant allele is reported in red.

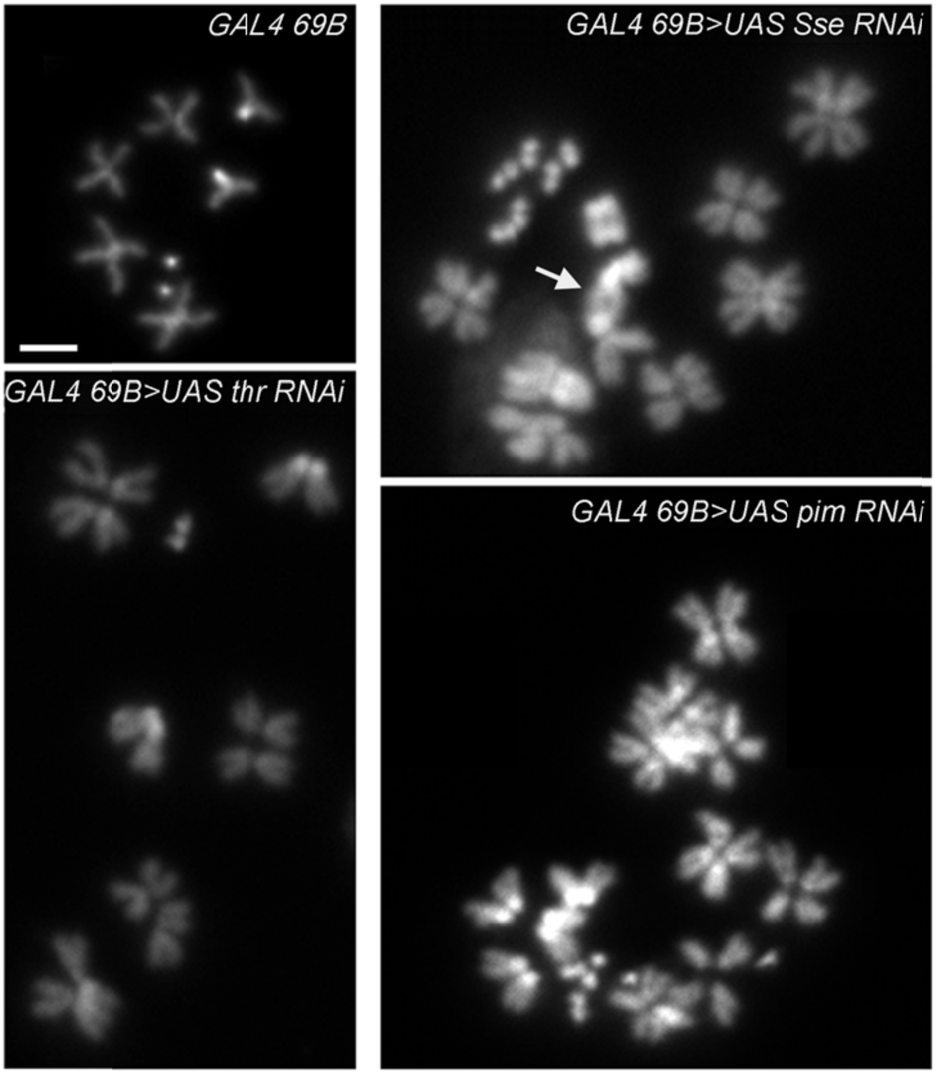

Supplementary Figure 4. Phenotypic analysis of *Sse* RNAi brain cells. DAPI stained colchicine-treated metaphases from different RNAi lines. Note that *Sse* RNAi cells display endoreduplication and TFs, whereas *pim* or *thr* RNAi cells exhibit endoreduplicated cells but not TFs. Scale bar, 5 µm.

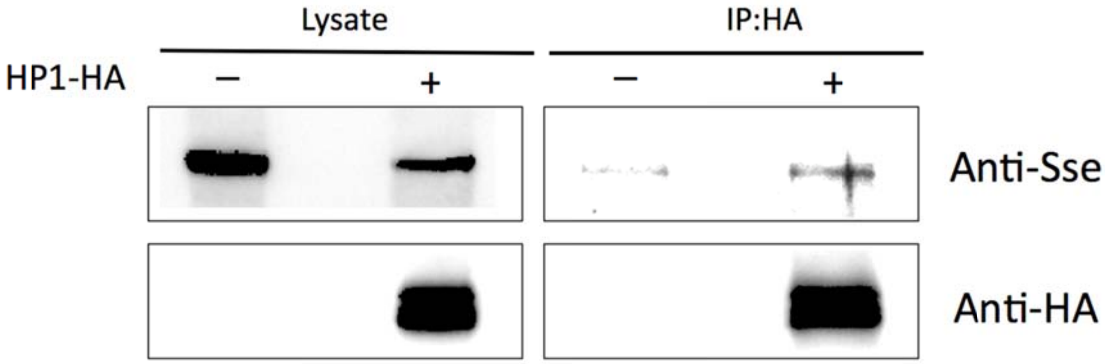

Supplementary Figure 5. HP1 interacts with endogenous Sse. Immunoprecipitation from HP1-HA expressing S2 extracts with an anti-HA affinity matrix. Note that HP1-HA is able to precipitate the endogenous Sse that was detected by using an anti Separase antibody. As a negative control, immunoprecipitation was performed on extracts from untransfected S2 cells. Lysate =10% of total protein extracts

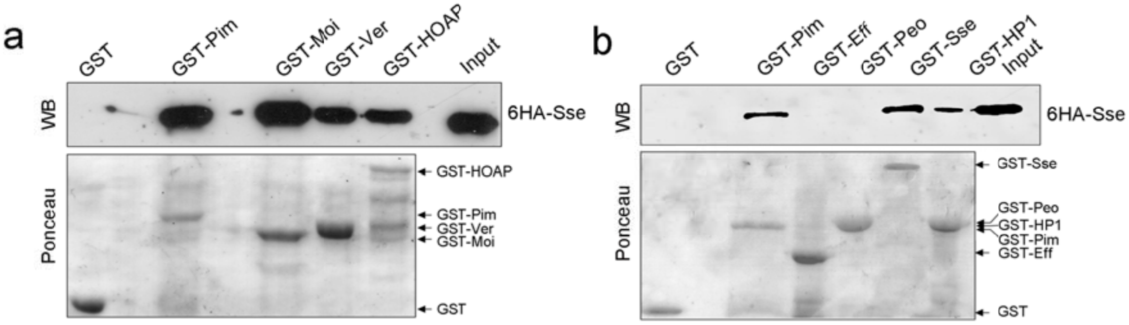

Supplementary Figure 6. Analysis of the physical interactions between Sse and telomeric proteins. (a) Bacterially expressed GST-tagged Moi, Ver and HOAP precipitate HA-Sse from larval brain extracts. (b) Bacterially expressed GST-HP1 and GST-Pim, but not GST-Eff or GST-Peo, precipitate HA-Sse from larval brain extract. Note that GST-Sse precipitates HA-Sse, very likely through its interactors. GST-Pim has been used as a positive control for Sse interaction in both (a) and (b). Input= 10% of total protein extracts.

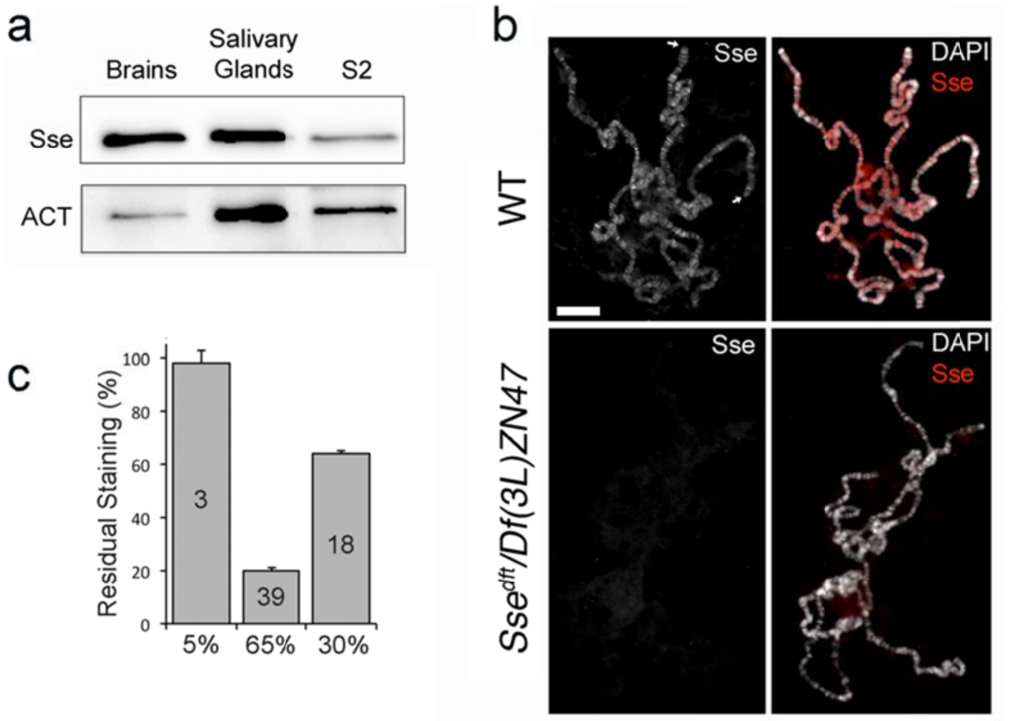

Supplementary Figure 7. Separase localization on polytene chromosomes. (a) Western Blot from larval brains, salivary glands and S2 cell extracts. (b) Wild-type (WT) and *Sse*^{dft}*/Df(3L)ZN47* mutant polytene chromosomes stained with Sse and with DAPI+Sse (red). Note that Sse localizes to many euchromatic bands and in some cases to telomeres (white arrows). (c) Quantification of Sse intensity in *Sse*^{dft} mutant glands showing the out of 60 mutant polytene nuclei 3 (5%), 39 (65%) and 18 (30%) exhibit 0, 80% and 35% reduction of Sse staining (with respect to wild-type, respectively. The signal quantification has been performed using the ImageJ software (National Institute of Mental Health, Bethesda, Maryland, USA). Oregon R has been used as wild-type. Scale bar, 20 µm.

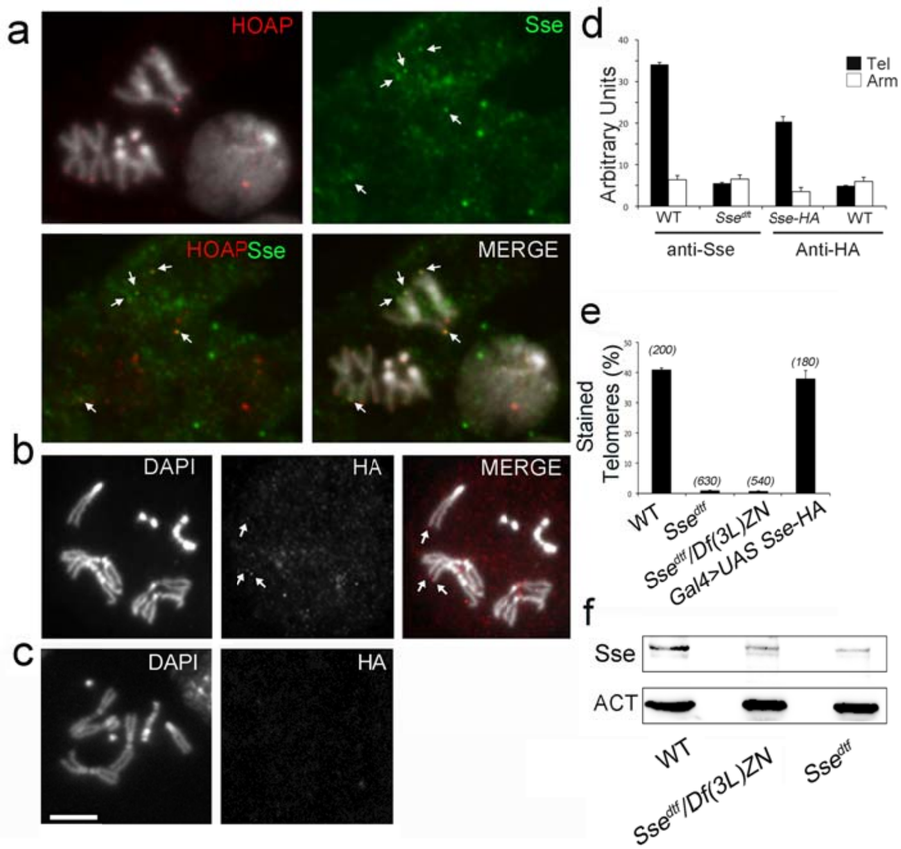

Supplementary Figure 8. Mitotic localization of Sse. a) Wild-type female metaphase stained for HOAP (red) and Sse (green). Note that Sse is enriched at most chromosome telomeres (arrow), where it co-localizes with HOAP (yellow spots in the merge). b) A male metaphase from a HA-Sse expressing brain immunostained with an anti-HA antibody shows an Sse enrichment at telomeres (arrows). c) Anti-HA staining of a wild-type male metaphase that does not express HA-Sse. Note the absence of staining. d) Quantification of Sse telomeric signals with anti-Sse and anti-HA antibodies used in a-c. Note that Sse is enriched at mitotic chromosome ends. e) Frequency of Sse stained telomeres in wild type, *Sse* mutant, and HA-Sse expressing brain cells; the numbers between parentheses indicate the numbers of telomeres examined. f) Western blot from *Sse*^{dft} homozygous and hemizygous brain extracts showing a substantial reduction of Sse levels in the mutants. Actin has been used as a loading control. Scale bar, 5 µm.

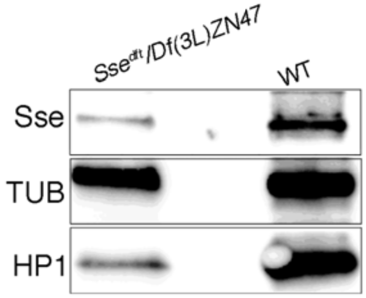

Supplementary Figure 9. Reduction of HP1 in *Sse^{dft}/Df(3L)ZN47* hemizygotes. Western blot has been performed from larval brain extracts. Tubulin (TUB) has been used as a loading control.

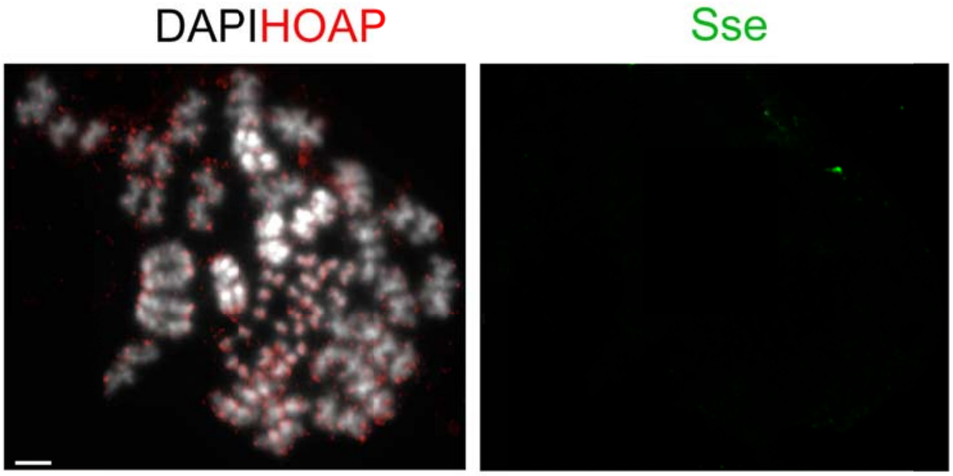

Supplementary Figure 10. **Sse is not required for HOAP localization**. *Sse* mutant metaphase showing a normal localization of telomeric HOAP (red). Note the lack of Sse staining in the right panel. Scale bar, 5 µm.

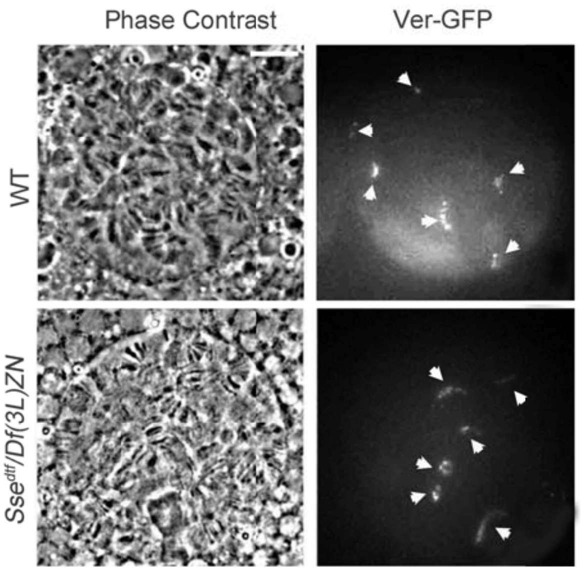

Supplementary Figure 11. Ver localization is not affected in *Sse* mutants. Ver-GFP-expressing larvae exhibit six discrete fluorescent signals (white arrows) on polytene chromosome telomeres[23]. Polytene chromosomes of *Sse* mutants that also express Ver-GFP display a Ver localization pattern which is undistinguishable from WT. Scale bar, 10 µm

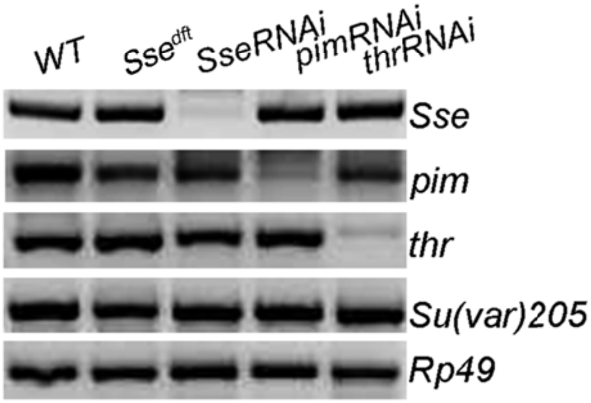

Supplementary Figure 12. **RT-PCR analysis of control and mutant brains for the expression of *Sse, pim, thr* and *Su(var)205* (HP1)**. Note that the *Su(var)205* transcript levels are not affected by *Sse* mutations. *Rp49* has been used as a loading control.

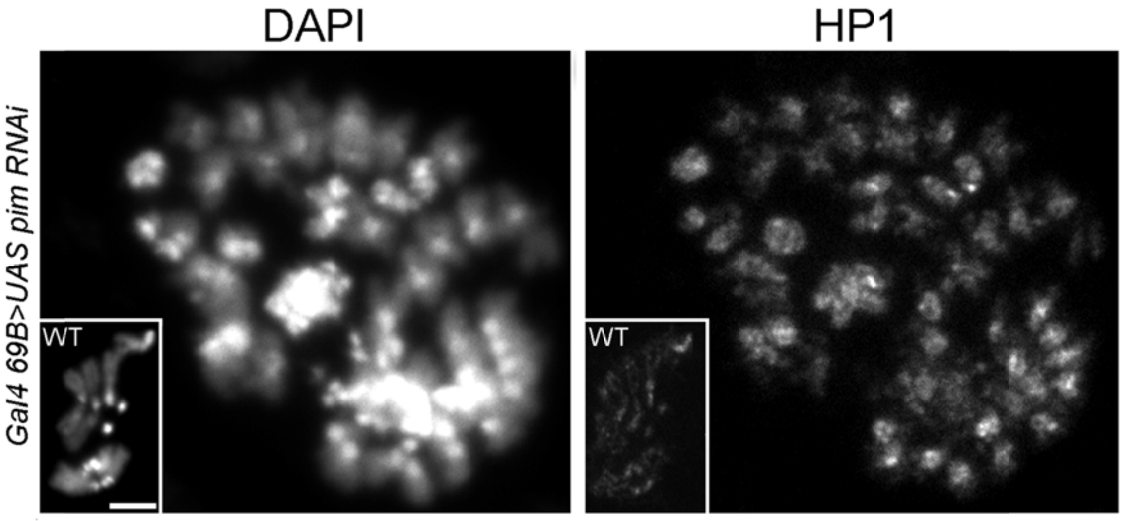

Supplementary Figure 13. Pim is not required for HP1 localization. Wild type (inset) and *69B GAL4>pim RNAi* mitotic chromosomes stained with DAPI and anti-HP1 antibody. Note that loss of Pim does not affect the overall HP1 localization. Scale bar, 5 µm.

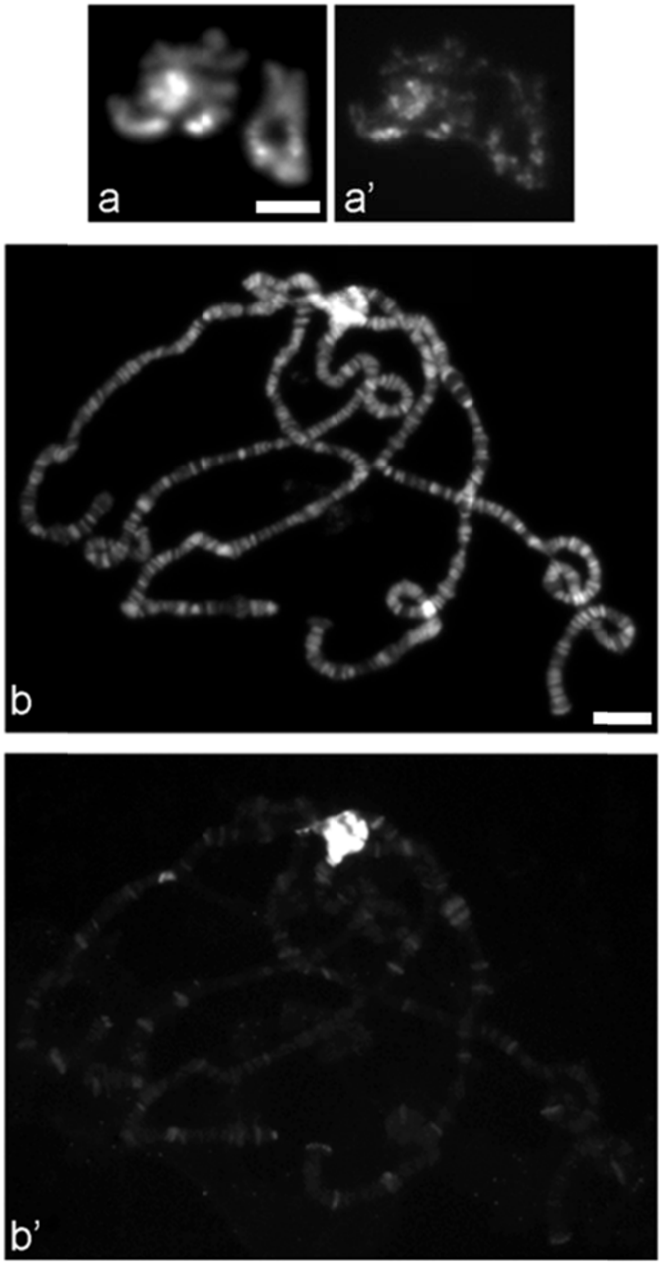

Supplementary Figure 14. Sse-HA rescues *Sse*^{dft} phenotypes*.* Mitotic (a,a' scale bar, 5 µm) and polytene (b, b' Scale bar, 20 µm) chromosomes from *Gal4>Sse-HA, Sse*^{dft} mutant brains stained with DAPI (a, b) and anti-HP1 (a', b'). Note the absence of endoreduplication in mitotic metaphases and the normal HP1 localization on both types of chromosomes.

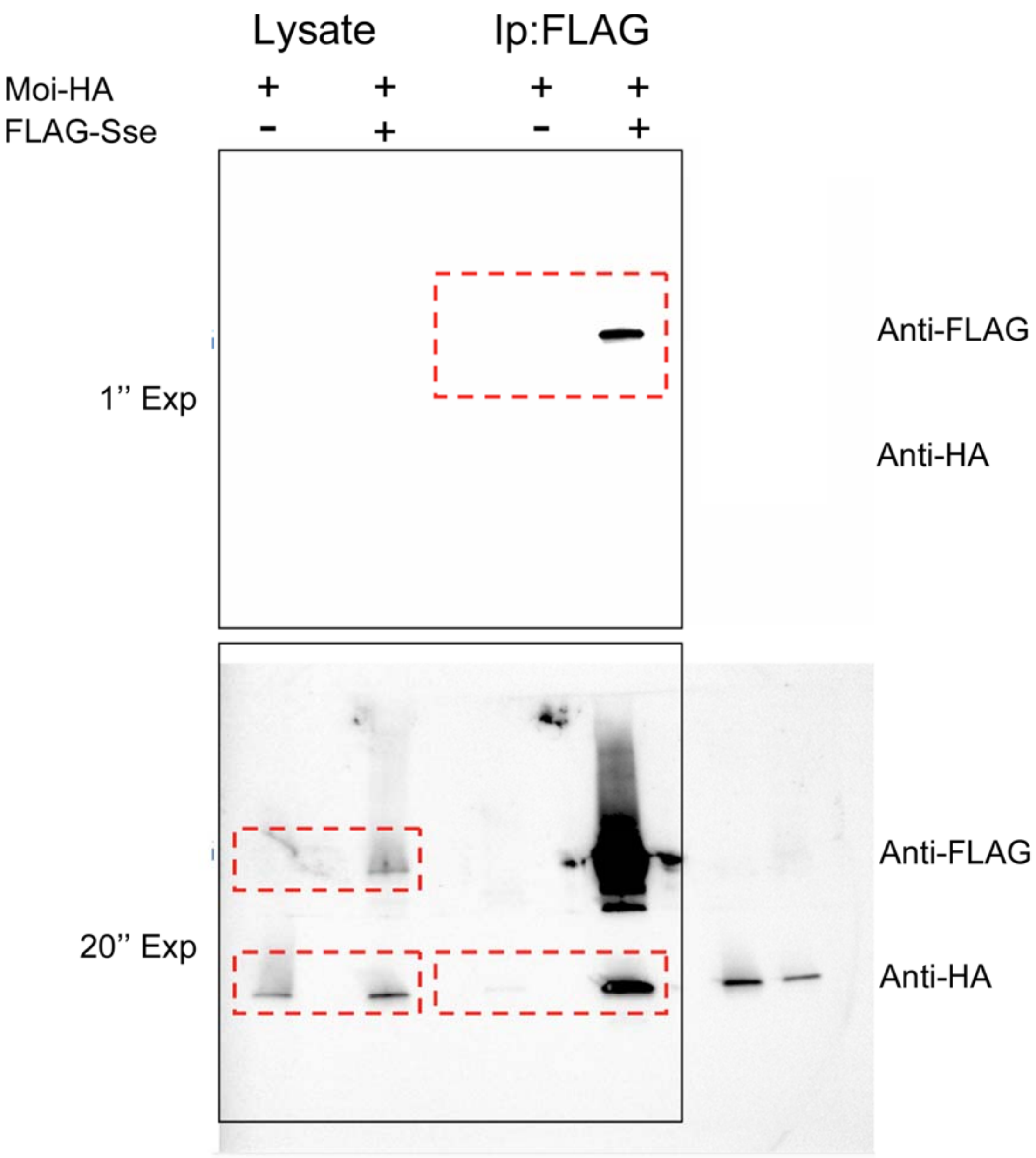

Supplementary Figure 15. Uncropped scan of WB shown in Figure 2b. Red rectangles refer to the sections used for panels of Figure 2b.

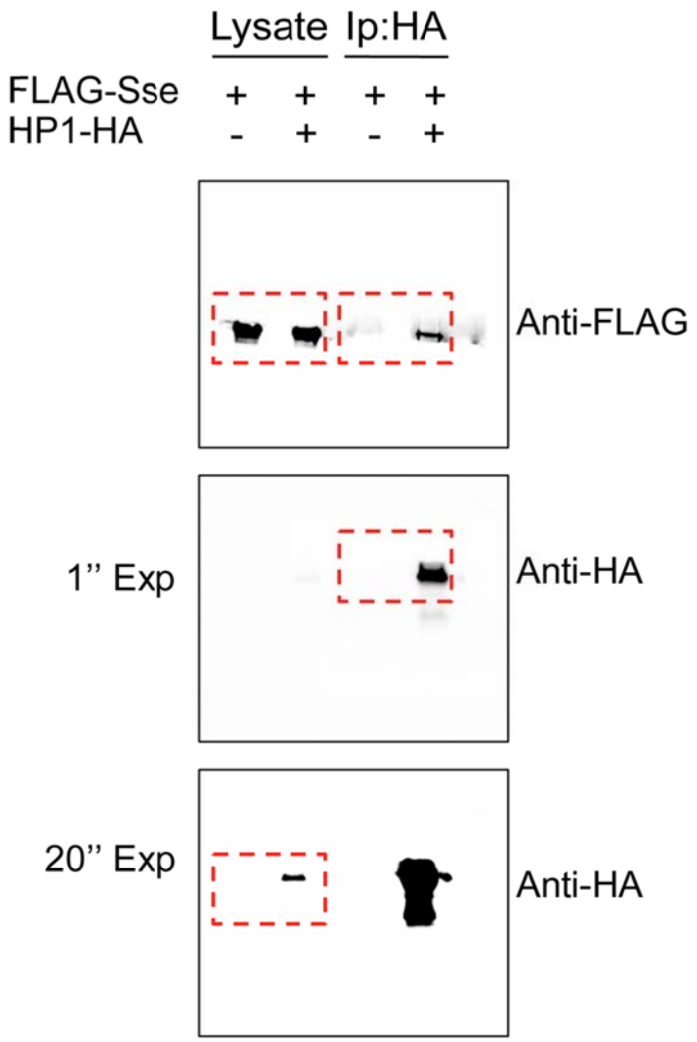

Supplementary Figure 16. Uncropped scan of WB shown in Figure 2e. Red rectangles refer to the sections used for panels of Figure 2e.

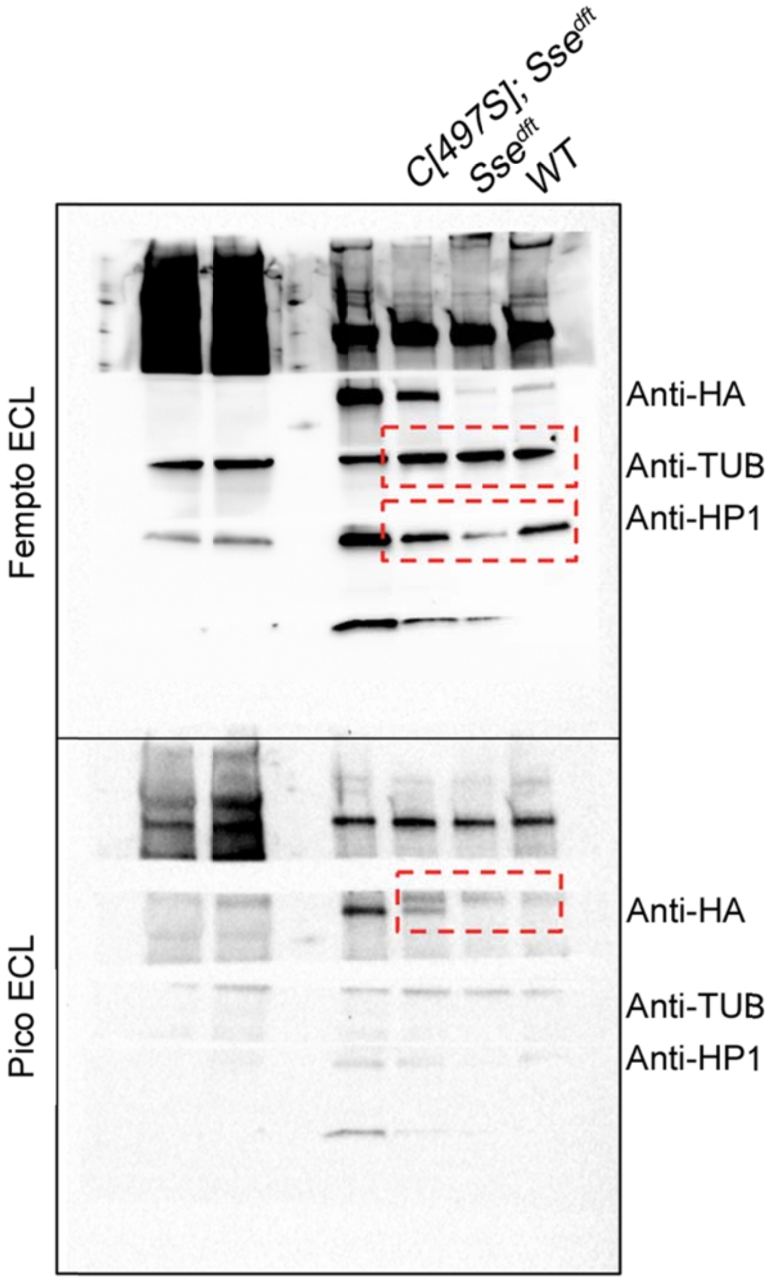

Supplementary Figure 17. Uncropped scan of WB shown in Figure 4i. Red rectangles refer to the sections used for panels of Figure 4i.

Supplementary Table

Genotype	Total Cells	Chromosome Content								DTA/TC (average)
		8		***16-32***		***32-64***		***>64***		
		PL (%)	DTA/TC	PL (%)	DTA/TC	PL (%)	DTA/TC	PL (%)	DTA/TC	
Sse^{dft}	250	14.0	0.33	50.0	0.43	24.0	0.33	12.0	0.32	0.35
Sse^{dft}*/l(3)13m-281*	260	4.7	0.30	59.5	0.42	25.0	0.35	13.0	0.31	0.34
Sse^{dft}*/Df(3L)ZN47*	230	12.0	0.27	54.0	0.51	19.0	0.40	15.9	0.26	0.36
[a]*Sse*^{dft}	50	10.0	0.28	56.0	0.52	26.0	0.46	8.0	0.35	0.40
[RFP-HP1] Sse^{dft}	80	20.0	0.02	47.0	0.05	25.0	0.06	8.0	0.04	0.04
UAS Sse^{C497S} *Sse*^{dft}	45	24.4	0.31	44.4	0.49	20.0	0.43	11.1	0.37	0.40
UAS Sse RNAi	108	88	0.04	12	0.04	-	-	-	-	0.04

Supplementary Table 1. Frequency of polyploidy (PL) and DTA/TC in different *Sse* mutant combinations or *SseRNAi.* Both frequencies are close to 0 in wild-type Oregon R cells (not indicated). [a] This value refers to *Sse*^{dft} mutant cells examined for the experiments outlined in Figure 4j and 4k.
